# Comparison in gene expression of secretory human endometrium using laser microdissection

**DOI:** 10.1186/1477-7827-2-66

**Published:** 2004-09-17

**Authors:** Atsushi Yanaihara, Yukiko Otsuka, Shinji Iwasaki, Keiko Koide, Tadateru Aida, Takashi Okai

**Affiliations:** 1 Department of Obstetrics and Gynecology, Showa University School of Medicine, Hatanodai, Shinagawa-ku, Tokyo, Japan

## Abstract

**Background:**

The endometrium prepares for implantation under the control of steroid hormones. It has been suggested that there are complicated interactions between the epithelium and stroma in the endometrium during menstrual cycle. In this study, we demonstrate a difference in gene expression between the epithelial and stromal areas of the secretory human endometrium using microdissection and macroarray technique.

**Methods:**

The epithelial and stromal areas were microdissected from the human endometrium during the secretory phase. RNA was extracted and amplified by PCR. Macroarray analysis of nearly 1000 human genes was carried out in this study. Some genes identified by macroarray analysis were verified using real-time PCR.

**Results:**

In this study, changes in expression <2.5-fold in three samples were excluded. A total of 28 genes displayed changes in expression from array data. Fifteen genes were strongly expressed in the epithelial areas, while 13 genes were strongly expressed in the stromal areas. The strongly expressed genes in the epithelial areas with a changes >5-fold were WAP four-disulfide core domain 2 (44.1 fold), matrix metalloproteinase 7 (40.1 fold), homeo box B5 (19.8 fold), msh homeo box homolog (18.8 fold), homeo box B7 (12.7 fold) and protein kinase C, theta (6.4 fold). On the other hand, decorin (55.6 fold), discoidin domain receptor member 2 (17.3 fold), tissue inhibitor of metalloproteinase 1 (9 fold), ribosomal protein S3A (6.3 fold), and tyrosine kinase with immunoglobulin and epidermal growth factor homology domains (5.2 fold) were strongly expressed in the stromal areas. WAP four-disulfide core domain 2 (19.4 fold), matrix metalloproteinase 7 (9.7-fold), decorin (16.3-fold) and tissue inhibitor of metalloproteinase 1 (7.2-fold) were verified by real-time PCR.

**Conclusions:**

Some of the genes we identified with differential expression are related to the immune system. These results are telling us the new information for understanding the secretory human endometrium.

## Introduction

Many studies have sought to understand the mechanism of implantation. Recently, the rate of pregnancy in the in vitro fertilization and embryo transfer (IVF-ET) cycle has declined, and this has been attributed to a decrease in the rate of implantation. 

The recently developed laser microdissection method has gained widespread use throughout the research field. Information about cells can be determined without contamination by using this method. Moreover, with the macroarray technique, which was already widely used for this purpose, the profiling of the gene expression of specific cells types has become possible. Torres et al. have already reported differences in gene expression between cell types or regions within the monkey endometrium using laser microdissection and differential display [1,2]. 

Identification of cell-specific proteins, which are expressed in the endometrium during the secretory phase, has been performed using a multi-disciplinary approach in the same trial. IGF-II mRNA is expressed in the mid-to-late secretory phase and in early pregnancy [3]. During decidualization, interstitial collagen in the mouse endometrium increases [4]. Collagen IV and laminin reactivity increased in the basal lamina and underlying subepithelial stroma as pregnancy proceeds [5]. The most abundant expression of IL-15 mRNA during the menstrual cycle is observed in the midsecretory phase [6]. Leukaemia inhibitory factor (LIF) is known as an indispensable factor for implantation and is expressed in the glandular epithelium at the time of implantation in human endometrium [7-9]. 

In this study, we demonstrate differential in gene expression between the epithelial and stromal areas obtained from secretory human endometrium using laser microdissection and the macroarray method. Confirmation of differential expression of candidate genes was performed by real-time PCR. 

## Materials and Methods

### Materials

Human endometrium was obtained from 8 patients (25–38 years old) with normal menstrual cycles (28~30 days) during the mid secretory phase. These patients had had at least one intrauterine pregnancy in the past (3 patients for Microarray analysis, 5 patients for real-time PCR). Part of the endometrial biopsy was obtained with a curetting technique. The day of the menstrual cycle was determined by the patient's history, plasma progesterone levels (9.8~17.3 ng/ml) and the histological criteria of Noyes et al [10]. These patients did not receive any hormonal therapy. Informed consent was obtained from all patients who participated in this study. The Institutional Review Boards of Showa University approved the use of human subjects and the procedures.

### Methods

#### Laser Microdissection and RNA extraction

**Figure 1:**
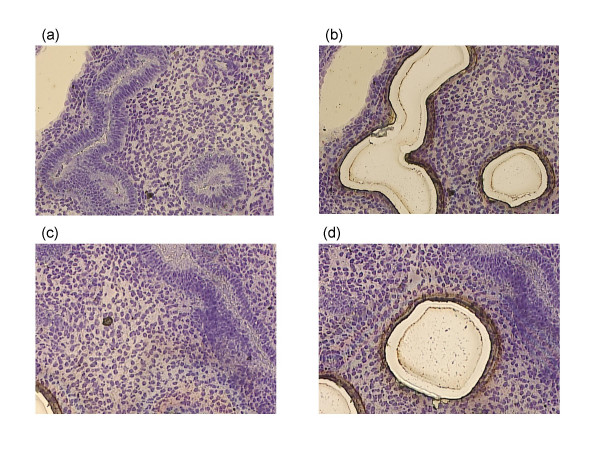
Human endometrial epithelial areas (a-b) and stromal areas (c-d) were laser-microdissected. (200×)

The endometrium was embedded in OCT compound and frozen immediately in isopentane that had been cooled in the liquid nitrogen. This freezing block was sliced by a cryomicrotome at 8 μm thickness. Frozen sections were fixed in 100% methanol for 3 min and stained with 1% toluidine blue. The section was laser-microdissected by the PALM MicroBeam system (PALM Microlaser. Technologies A.G.) for epithelial and stromal areas and collected in a small tube (Fig 1a,1b,1c,1d). Approximately 30–50 sections were laser-microdissected. Contamination with non-target components was monitored morphologically. Total RNA was extracted from the tissue section using the acid guanidinium-phenol-chloroform method [11]. 

### Macroarray

The RNAs obtained were synthesized from cDNA using a modified oligo (dT) primer and the BD SMART™ PCR cDNA Synthesis Kit (BD Biosciences Clontech, Palo Alto, CA). cDNA was PCR amplified for 24–29 cycles according to the user manual. (BD Atlas™ SMART™ Probe amplification Kit (BD Biosciences Clontech, Palo Alto, CA)). 550 ng of cDNA sample was labeled with α-^32^P dCTP (3000 Ci/mmol) using a randam primer. Labeled probes were hybridized to a nylon array (BD Atlas™ Nylon cDNA Expression Arrays, Human 1.2 Array (BD Biosciences Clontech, Palo Alto, CA)) in ExpressHyb solution at 68°C overnight. After hybridization, the nylon membrane was washed with 2 × standard saline citrate (SSC) + 1% sodium dodecyl sulphate (SDS) (WAKO Pure Chemical Ltd, Japan) once, twice with 1.0 × SSC + 0.5% SDS at 68°C [12,13]. The membrane was exposed to a phosphor screen (Fujifilm, Japan) for 24 hours and scanned using a STORM 830 Scanner and IMAGEQUANT 4.1-J (Molecular Dynamics). Hybridization signal intensities for individual genes were a subtracted from the background and normalized to the signals for GAPDH and the beta-actin gene, respectively, using AIS (Analytical Imaging Station) Array™ (IMAGING Research INC.). Each normalized a gene expression signal the epithelial and stromal areas was compared, and was automatically calculated as a ratio [14,15]. In this study, a change in expression <2.5-fold in all three samples was excluded.

#### Real-time PCR

RNA was reverse transcribed using oligo (dT) primers by TaKaRa RNA PCR Kit (AMV) Ver 2.1 (TAKARA BIO INC, Shiga, Japan) according to the manufacturer's instructions. PCR was performed using the ABI PRISM 7700 Sequence Detection System. TaqMan Universal PCR MasterMix and Assays-on-Demand Gene Expression probes (Applied Biosystems) were used for the PCR step (Assay ID for MMP7; Hs00159163 m1, WFDC2; Hs00707910 s1, TIMP1; Hs00171558 m1 Decorin; Hs00370385 m1). Primer sequences are not publicly available, although their validity has been established by the manufacturer. The expression values obtained were normalized against those from the control human GAPDH [16]. Statistical significance was determined by the Wilcoxon test and defined as p < 0.05.

## Results

### Microdissection and Microarray

Secretory endometrium was collected from 8 patients (3 patients for Microarray, 5 patients for real-time PCR). Each sample was carefully dissected by laser microdissection for epithelial and stromal areas (Fig 1a to 1d).

Total RNA was extracted and subjected to macroarray with nearly 1000 genes on the nylon membrane. Fifteen genes were strongly expressed in the epithelial areas (Table 1), while 13 genes were strongly expressed in the stromal areas (Table 2). Mean values are shown in Tables 1 and 2. Genes strongly expressed in the epithelial areas that increased >5-fold in expression included WAP four-disulfide core domain 2 (WFDC2), matrix metalloproteinase 7 (MMP7), homeo box B5, msh homeo box homolog, homeo box B7 and protein kinase C, theta (PKC theta). On the other hand, decorin, discoidin domain receptor member 2 (DDR2), tissue inhibitor of metalloproteinase 1 (TIMP1), ribosomal protein S3A, and tyrosine kinase with immunoglobulin and epidermal growth factor homology domains (Tie1) were strongly expressed in the stromal areas. 

**Table 1:** Expressed gene list in epithelial areas.

**Ratio**	**GENE BANK**	**LOCUS LINK**	**Gene Name**	**Classifications**
44.1	X63187	10406	WAP four-disulfide core domain 2	inhibitors of proteases
40.1	X07819	4316	matrix metalloproteinase 7	metalloproteinases
19.8	M92299	3215	homeo box B5	CDK inhibitors
18.8	M97676	4487	msh homeo box homolog 1 (Drosophila)	transcription activators and repressors
12.7	M16937	3217	homeo box B7	transcription activators and repressors
6.4	L07032	5588	protein kinase C, theta	intracellular kinase network members
4.8	X59798	595	cyclin D1	cyclins
4.5	M97796	3398	inhibitor of DNA binding 2, dominant negative helix-loop-helix protein	transcription activators and repressors
3.9	X02920	5265	serine proteinase inhibitor, clade A	inhibitors of proteases
3.8	D14520	688	Kruppel-like factor 5	basic transcription factors
2.9	U24166	22919	microtubule-associated protein, RP/EB family, member 1	adaptors and receptor-associated proteins
2.9	X67055	3699	pre-alpha (globulin) inhibitor	inhibitors of proteases
2.8	U26710	868	Cas-Br-M (murine) ectropic retroviral transforming sequence b	adaptors and receptor-associated proteins
2.7	D45132	7799	PR domain containing 2, with ZNF domain	transcription activators and repressors
2.5	AF059244	10047	cystatin 8	inhibitors of proteases

Ratio shows average of 3 samples

**Table 2:** Expressed gene list in stromal areas

**Ratio**	**GENE BANK**	**LOCUS LINK**	**Gene Name**	**Classifications**
55.6	M14219	1634	decorin	cell surface antigens
17.3	X74764	4921	discoidin domain receptor family, member 2	intracellular transducers
9	X03124	7076	tissue inhibitor of metalloproteinase 1	extracellular secreted proteins
6.3	M77234	6189	ribosomal protein S3A	ribosomal proteins
5.2	X60957	7075	tyrosine kinase with immunoglobulin and epidermal growth factor homology domains	intracellular transducers
4.9	M57399	5764	pleiotrophin (heparin binding growth factor 8)	growth factors
4.8	M62424	2149	coagulation factor II (thrombin) receptor	intracellular transducers
3.5	S40706	1649	DNA-damage-inducible transcript 3	other apoptosis-associated proteins
3.3	M81757	6223	ribosomal protein S19	other cell cycle proteins
2.8	J00123	5179	proenkephalin	neuropeptides
2.8	M15395	3689	integrin, beta 2	major histocompatibility complex
2.8	U32944	8655	dynein, cytoplasmic, light polypeptide	other apoptosis-associated proteins
2.6	D15057	1603	defender against cell death 1	other apoptosis-associated proteins

Ratio shows average of 3 samples

### Real-time PCR

Real-time PCR (Taqman analysis) was used to verify the changes in expression of certain candidate genes that are highly expressed in the array. Five samples were used for this study. WFDC2 and MMP7, which are both strongly expressed in the epithelial areas and decorin and TIMP1, which are both strongly expressed in the stromal areas by the cDNA array were chosen for verification. Each value was corrected for differences in loading relative to GAPDH mRNA expression. 

**Figure 2:**
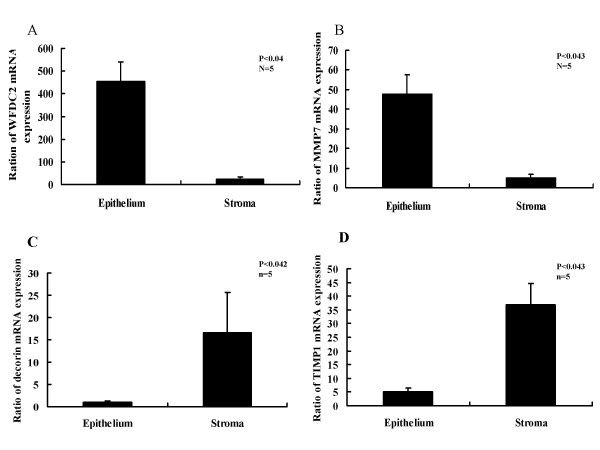
**A: **WFDC2 mRNA expression in the secretory phase of the endometrium was determined by real-time PCR (n = 5). Values were normalized to GAPDH mRNA expression. Epithelial areas WFDC2 mRNA expression is shown relative to that of the stromal areas. The mean change is 19.4-fold. Statistical analysis was carried out using Wilcoxon test. **B: **MMP7 mRNA expression in the secretory phase of the endometrium was determined by real-time PCR (n = 5). Values are normalized to GAPDH mRNA expression. Epithelial areas MMP7 mRNA expression is shown relative to that of the stromal areas. The mean change is 9.7-fold. Statistical analysis was carried out using Wilcoxon test. **C: **Decorin mRNA expression in the secretory phase of the endometrium was determined by real-time PCR (n = 5). Values were normalized to GAPDH mRNA expression for each sample. Stromal areas decorin mRNA expression is shown relative to that of the epithelial areas. The mean change is 16.3-fold. Statistical analysis was carried out using Wilcoxon test. **D: **TIMP1 mRNA expression in the secretory phase of the endometrium was determined by real-time PCR (n = 5). Values are normalized to GAPDH mRNA expression for each sample. Stromal areas TIMP1 mRNA expression is shown relative to that of the epithelial areas. The mean change is 7.2-fold. Statistical analysis was carried out using Wilcoxon test.

WFDC2 and MMP7 mRNA expression increased by 9.4- and 9.7-fold, respectively, compared to that of stromal cells. Decorin and TIMP1 mRNA expression increased by 16.3-and 7.2-fold, respectively, in stromal cells compared to that of epithelial cells. Statistically significant changes in expression of these genes were observed (p < 0.05) (Fig 2A,2B,2C and 2D).

## Discussion

To date, various methods have been used for understanding the function of the endometrium. It is a well-known fact that epithelial cells and stromal cells in the endometrium play specific roles and are influenced by steroid hormones. However, it is very difficult to understand the molecular composition of each cell type as a function of time during the menstrual cycle. One of the problems of a cell culture experiment is that separation cultivation makes changes the composition of the cells. This is especially true as these cells are influenced by neighboring cells *in vivo*. It has recently become possible to acquire the information about the cell by the microdissection method. 

In this study, laser microdissection was used to isolate epithelial and stromal areas from the human endometrium. RNA was amplified by PCR and global gene expression was demonstrated by cDNA macroarray. 

Twenty-eight genes were identified in this study. These constitute only 2.8% of the 1000 genes on the array. Although this seems to be a small number, these genes were expressed at least 2.5-fold greater in all three samples and normalized to two house keeping genes. A similar percentage (1.2–5.8%) of genes with differential expression were reported using array analysis [17-19]. However, included genes below the 2.5-fold that we established as a criterion for inclusion in this study should be considered.

Recently, some papers focused on endometrial gene expression have been reported. However, lots of them were compared between phases in the menstrual cycle. While Okulicz et al. demonstrated a difference in the gene expression between cell compartments in the monkey endometrium, the genes they identified are not the same as ours [1,2]. One of the reasons for this is because they tried to find new genes using differential display RT-PCR. 

Fifteen of 1000 genes were strongly expressed in the epithelial areas compared to the stromal areas. Of these, WFDC2 and MMP 7 were strongly expressed in the epithelial areas as confirmed by real-time PCR. WFDC2 was originally described as an epididymis-specific protein is expressed in a number of normal human tissues. A possible role for this gene in sperm maturation is indicated by amino acid similarities to extracellular proteinase inhibitors of genital tract mucous secretions [20]. Although WFDC2 has been recently reported in the secretory endometrium of monkey, this is the first report of its localization in the epithelium of the human endometrium [21]. However, the physiological role of this gene in the endometrium is presently unknown. Baboon endometrial epithelia express MMP 7 was reported by Cox et al [22]. The highest expression of MMP 7 occurred on day 7 of pregnancy in the rat uterus [23,24] and has been reported to have close associations with tumor invasion and metastasis [25,26].

HOXB gene induction is related to the immune system, and is specifically associated with IL-2-induced NK cell proliferation [27,28]. Although Hox-7 is reported to be in human cervical tumor tissue [29], this is the first report or its localization in human endometrium. Msh genes play a role in the regulation of cell-cell adhesion [30]. Friedmann et al. reported that regulated expression of homeobox genes Msx-1 and Msx-2 in mouse mammary gland development suggests a role in hormone action and epithelial-stromal interactions [31]. PKC theta cooperates with Vav1 to induce JNK activity in T-cells [32,33]. Take Catalano et al. report that JNK pathways are altered by RU486 which is an antiprogestins. PKC therefore seems to be important factor for control secretory endometrium [17].

Of the strongly expressed genes in the stromal aeas, decorin and TIMP1 gene expression were verified by real-time PCR. San Martin et al. reported the expression of decorin which is a leucine-rich proteoglycan in the mouse uterine and suggested it localized in the undifferentiated interimplantation site stroma [34]. Some reports also demonstrated its presence in the human uterin cervix and myometrium, but not in human endometrium [35,36]. DDR2 is a new type of receptor tyrosine kinases, and is thought to be involved in the metastasis of some tumors. Its ligand is fibrillar collagen, which suggests a role in controlling celluar responses to the extracellular matrix [37]. The changes in the extracellular matrix may play an important role in implantation, in invasion of trophoblastic cells and in the maintenance of pregnancy [38]. Decidualized stromal cells stained strongly positive for TIMP-1 [39]. Matrix metalloproteinases and their endogenous inhibitors, tissue-specific inhibitors of matrix metalloproteinases, play key roles in the cyclic remodeling events that occur in the human endometrium in preparation for pregnancy [40]. Ribosomal protein S3A is through direct or indirect actions on B and T cells and cytokine secretion, could participate in the immunoregulatory processes that play a role in the balance of the Th1 and Th2 immune response [41]. It has been recently said that the ratio of Th1 to Th2 influences pregnancy. Ribosomal protein S3A may be an interesting factor for stromal cell research. In this study, these results show the fact that many of the genes, which are related to the immune system, are expressed in the endometrium during the mid secretory phase of the menstrual cycle. This time, however, differences in gene expression between cell compartments of the endometrium were considered. Interactions among cells are key factors in understanding endometrial function.
